# METABOLIC CONTROL AND BODY COMPOSITION OF CHILDREN AND ADOLESCENTS WITH PHENYLKETONURIA

**DOI:** 10.1590/1984-0462/2021/39/2020095

**Published:** 2021-02-24

**Authors:** Zeni Drubi Nogueira, Ney Boa-Sorte, Maria Efigênia de Queiroz Leite, Maria Betânia Pereira Toralles, Tatiana Amorim

**Affiliations:** a Universidade Federal da Bahia, Salvador, BA, Brazil.; b Universidade do Estado da Bahia, Salvador, BA, Brazil.; c Association of Parents and Friends of Intelectually Disabled Individuals (Associação de Pais e Amigos dos Excepcionais de Salvador - APAE), BA, Brazil.

**Keywords:** Metabolism, inborn errors, Phenylketonurias, Nutritional status, Body composition, Erros inatos do metabolismo, Fenilcetonúrias, Estado nutricional, Composição corporal

## Abstract

**Objective:**

To characterize metabolic control and verify whether it has any relation with socioeconomic, demographic, and body composition variables in children and adolescents with phenylketonuria (PKU) diagnosed in the neonatal period.

**Methods:**

This cohort study collected retrospective data of 53 phenylketonuric children and adolescents. Data on family income, housing, and mother’s age and schooling level were collected, and anthropometric measures of body composition and distribution were taken. All dosages of phenylalanine (Phe) from the last five years (2015-2019) were evaluated and classified regarding their adequacy (cutoffs: 0-12 years: 2-6 mg/dL; 12-19 years: 2-10 mg/dL). Adequate metabolic control was considered if ≥7%) of the dosages were within desired ranges.

**Results:**

The mean (±standard deviation) age in the last year was 10.1±4.6 years. Most of them were under 12 years old (33/53; 62.3%) and had the classic form of the disease (39/53; 73.6%). Better metabolic control was observed among adolescents (68.4 *versus* 51.4%; p=0.019). Overweight was found in 9/53 (17%) and higher serum Phe levels (p<0.001) were found in this group of patients. Metabolic control with 70% or more Phe level adequacy decreased along with the arm muscle area (AMA) (p_tendency_=0.042), being 70.0% among those with low reserve (low AMA), and 18.5% among those with excessive reserve (high AMA).

**Conclusions:**

Adequate metabolic control was observed in most patients. The findings suggest that, in this sample, the levels of phenylalanine may be related to changes in body composition.

## RESUMO

**Objetivo:**

Caracterizar o controle metabólico e verificar se existe relação entre ele, variáveis socioeconômicas, demográficas e composição corporal de crianças e adolescentes com fenilcetonúria (FNC) diagnosticada no período neonatal.

**Métodos:**

Coorte com coleta retrospectiva de dados de 53 crianças e adolescentes fenilcetonúricos. Foram coletados dados de renda familiar, moradia, idade e escolaridade materna e realizaram-se medidas antropométricas de composição e distribuição corporal. Todas as dosagens de fenilalanina (Fal) dos últimos cinco anos (2015-2019) foram avaliadas e classificadas quanto à adequação (cortes: 0-12 anos: 2-6 mg/dL; 12-19 anos: 2-10 mg/dL). A proporção de dosagens adequadas ≥70% foi considerada como controle metabólico adequado.

**Resultados:**

A média (±desvio padrão) de idade, no último ano, foi de 10,1±4,6 anos. A maioria tinha menos de 12 anos (33/53; 62,3%) e apresentava a forma clássica da doença (39/53; 73,6%). Observou-se melhor controle metabólico entre os adolescentes (68,4 *vs*. 51,4%; p=0,019). Excesso de peso foi encontrado em 9/53 (17%) e maiores níveis séricos de Fal foram descritos nesse grupo (p<0,001). O percentual de controle metabólico com 70% ou mais de adequação dos níveis de Fal foi decrescente de acordo com a área muscular do braço (AMB; p_tendência_=0,042), sendo de 70% entre os de baixa reserva (AMB reduzida) e de 18,5% entre os com excesso (AMB elevada).

**Conclusões:**

Observou-se controle metabólico adequado na maioria dos avaliados e os achados sugerem que, nesta amostra, os níveis de fenilalanina podem estar relacionados com alterações da composição corporal.

## INTRODUCTION

Phenylketonuria (PKU) is a metabolic disease resulting from the accumulation of blood phenylalanine (Phe), which crosses the blood-brain barrier, implying neurotoxicity, and is characterized by intellectual disability (ID) when not diagnosed and treated early.^1^ Treatment consists of a restricted diet of Phe for life, being effective to avoid neurocognitive sequelae, especially when started in the first days of life.^2^

The multidisciplinary team and the patient’s family have the challenge of providing means for adhering to the diet, which is extremely restrictive and has little variety of preparations.^3^ Studies show that adherence to the diet decreases with age, making follow-up more difficult for adolescents, adults and pregnant women.^4^

In addition to neurocognitive damage, phenylketonuric patients without metabolic control are at increased risk of being overweight,^5^ ^,^ ^6^ and girls are more likely to it.^7^ A previous study conducted at eight European centers suggested early monitoring and intervention to prevent and control overweight.^8^

When evaluating 30 phenylketonuric children and adolescents aged 5-18, a study associated increased body fat and reduced muscle mass with a diet rich in carbohydrates and low intake of fat and protein. In addition, inadequate metabolic control had a positive correlation with body fat.^5^ Similarly, among Dutch phenylketonuric individuals compared to healthy controls, the percentage of body fat was significantly higher, especially in 11-year-old girls or older, although without differences in body mass index for age (BMI/A).^9^ However, the authors considered the effect of Phe levels on the outcome studied to be inconclusive.^9^

In Brazil, research did not find differences in the frequency of overweight, fat percentage, and lean body mass when comparing 27 individuals aged 6-25 with PKU and healthy controls matched for age and sex. There was also no effect of metabolic control of serum Phe levels on body composition.^10^ These results were also found in Brazilian phenylketonuric adolescents from the Southeastern region, in which no association was found between adequate metabolic control and body fat percentage.^11^ However, Almeida et al. reported a positive correlation of Phe levels with body weight and age in Brazilian phenylketonuric adolescents.^12^

Given this and the absence of data that nutritionally characterize children and adolescents with PKU in Northeast Brazil, the present study aimed to characterize metabolic control and to verify whether there is a relationship between it, socioeconomic and demographic conditions, and body composition of children and adolescents with PKU diagnosed in their neonatal period.

## METHOD

Cohort study with retrospective data collection, carried out between March and October 2019. A total of 70 children and adolescents served at the Reference Service for Neonatal Screening (*Serviço de Referência em Triagem Neonatal* - SRTN) in Bahia State, *Apae* Salvador, aged 2-19, with a neonatal diagnosis of mild or classic PKU were eligible. After accepting to participate in research, 57 individuals were included. Of these, three were excluded for not allowing the measurement of anthropometric measurements and one for having congenital heart disease. The eligible individuals not included were: one for refusal and 12 for missing appointments scheduled during the study period.

All Phe dosages recorded in the medical records from January 2015 to October 2019 were used. Patients monitored at the SRTN perform serum Phe measurements in all consultations, and collections are requested in intervals between visits to the SRTN, which are performed at the health unit closest to the family’s residence and sent to the laboratory of Apae Salvador. Anthropometric data were obtained at the last consultation at the service, collected by a single researcher (ZDN) and used to calculate the studied indicators. Weight, height, arm circumference (AC), tricipital skinfold thickness (TST) and subscapular skinfold (SS), waist circumference (WC) and abdominal circumference (AbC) were measured. In addition, socioeconomic and demographic data (education and maternal age on the day of the consultation, family income, place of residence) were collected.

The anthropometric assessment indicators were calculated in Z score by the AnthroPlus^®^ program.^13^ Height for age (H/A) and BMI/H were evaluated for all individuals, according to recommended reference values.^14^ Body composition measures AC, TST, and SS,^15^sum of TST/SS,^16^ arm muscle area (AMA)^15^ and central adiposity, AbC and WC^17^ ^,^ ^18^ were classified according to the cutoff points previously described. The anthropometric indicators of BMI/H, AC, TST, SS, and TST+SS were grouped into three categories: low reserve/thinness, adequate/normal weight and excess, and WC and AbC were classified as adequate or increased.

The quantitative measurement of Phe was performed by the enzymatic colorimetric method, using the NeoLISA^®^PKU Intercientifica kit. All blood samples collected were analyzed by the SRTN laboratory. The classification of metabolic control considered adequate levels of serum Phe levels between 2-6 ­mg/­dL for children under 12 years old, and between 2-10 ­mg/­dL for those ≥12 years old.^19^

A percentage of adequacy of the metabolic control was proposed for classifying them as adequate or inadequate in the study period (five years). A minimum target of 70% of Phe dosages in the age-appropriate range was established as adequate. This criterion is adopted in the 2019-2020 Guidelines of the Brazilian Society of Diabetes, a chronic disease that also requires strict management of serum glucose levels.^20^ For comparative purposes, the same assessment was performed, targeting the number of dosages equal to or greater than 50% (metabolic control≥50%) and 90% (metabolic control≥90%) of the Phe dosages in the adequacy range.

The data were analyzed with Stata^®^, version 13.0. Descriptive statistics were used to characterize the studied variables. The variation in the averages of Phe dosages in the five years of analysis was represented by serial boxplots, stratified by year and age group. The mean levels (standard deviation) of serum Phe measurements were compared according to sociodemographic and anthropometric variables with the unpaired Student’s t test or analysis of variance (ANOVA), respectively, for two or three groups. *Post hoc* analyzes to assess intra-group differences were performed using the Bonferroni test. The adequacy of metabolic control (adequate/inadequate) was compared between groups using the chi-square test or Fisher’s exact test. Chi-square linear trend was used to assess trends in the case of variables with three categories. Values of p<0.05 were considered significant.

The present study was approved by the Research Ethics Committee of the Institute of Health Sciences, Universidade Federal da Bahia, opinion No. 3.181.463/2019.

## RESULTS

A total of 53 individuals aged 2-19 were studied, with a mean (standard deviation) of 10.1 (4.6) years, with 33 of them (62.3%) under 12 years old. Most were girls (34/53; 64.1%), and 39 (73.6%) had classical PKU.

**Graph 1:**
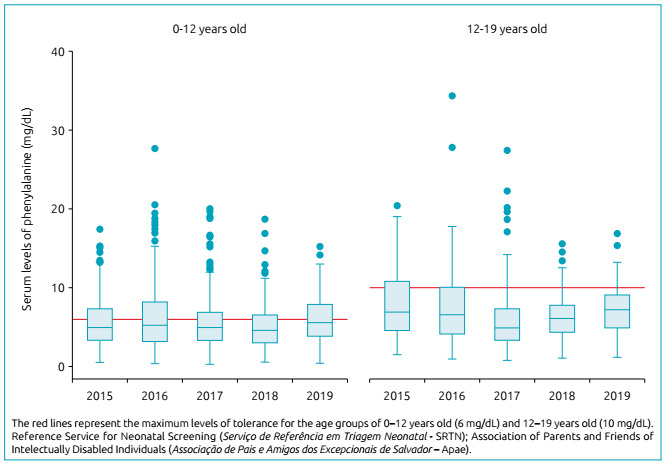
Serial boxplots of 2,242 serum levels of phenylalanine (in mg/dL) over the last five years of 53 children and adolescents with phenylketonuria. SRTN/Apae Salvador, 2019.

2,242 serum Phe measurements were obtained, with a mean (standard deviation) of 8.4 (2.1) exams/patient/year, with no difference between groups by age group (<12: 8.3 [2.0] *versus* ≥12: 8.7 [2.2]; p=0.525). The median ­(p25-­p75) of serum Phe levels fluctuated little in the study period among age groups (Graph 1), but it was higher among adolescents aged 12-19 (p<0.001). The global percentage of adequacy (minimum-maximum) observed corresponded to 57.8% (2.5-100), being higher in the 12-19 age group (68.4 *versus* 51.4%; p=0.019), and with better metabolic control in this group (p=0.003; Table 1).

**Table 1:** Characterization of serum phenylalanine dosages, and percentage of children and adolescents with dosages in the appropriate range for values ≥50, 70, and 90%, respectively, of 53 children and adolescents with PKU, stratified by age group. SRTN/Apae Salvador, 2019.

	All	0-12 years old	12-19 years old	p-value
	n (%)	n (%)	n (%)	
Phenylalanine (mg/dL)				
Number of dosages	2.242	1.704	538	<0.001
Mean (SD)	6.0 (3.5)	5.6 (3.3)	7.2 (4.0)	
Median (p25-p75)	5.4 (3.5-7.7)	5.0 (3.3-7.2)	6.8 (4.5-9.0)	
Classification				
Below the threshold	174 (7.8)	161 (9.4)	13 (2.4)	<0.001
On the threshold	1.348 (60.1)	920 (54.0)	428 (79.6)	
Above the threshold	720 (32.1)	623 (36.6)	97 (18.0)	
Adequate dosages (%)				
Average	57.8	51.4	68.4	0.019
Min-Max	2.5-100	4.5-92.2	2.5-100.0	
Metabolic control 50%				
Yes	34 (64.2)	18 (54.5)	16 (80.0)	0.080^a^
No	19 (35.8)	15 (45.5)	04 (20.0)	
Metabolic control 70%				
Yes	21 (39.6)	08 (24.2)	13 (65.0)	0.003
No	32 (60.4)	25 (75.8)	07 (35.0)	
Metabolic control 90%				
Yes	04 (7.6)	01 (3.0)	03 (15.0)	0.145^a^
No	49 (92.4)	32 (97.0)	17 (85.0)	

PKU: phenylketonuria; SRTN: Reference Service for Neonatal Screening (*Serviço de Referência em Triagem Neonatal*); Apae: Association of Parents and Friends of Intelectually Disabled Individuals (*Associação de Pais e Amigos dos Excepcionais de Salvador*); SD: standard deviation; ^a^Fisher’s exact test.

The group with lower family income had a higher mean [standard deviation] of Phe serum (6.72[3.66]mg/dL *versus* 5.63[3.68]mg/dL; p<0.001), but with no difference in the adequacy of metabolic control (26.1 *versus* 45.8%; p=0.159, Table 2). Higher serum levels of Phe were also observed among mothers with more schooling (p=0.006) and over 40 years old (p<0.001), but were not associated with worse metabolic control (Table 2).

**Table 2:** Description of serum phenylalanine levels (in mg/dL) and adequate metabolic control ≥70% in 53 children and adolescents with phenylketonuria, stratified by sex, disease classification, and demographic and socioeconomic variables. SRTN Apae Salvador, 2019.

	All Phe dosages (mg/dL)						Adequate metabolic control ≥70%					
	n (%)	Mean (SD)	p-value	Age range (years old)				n (%)	p-value	Age range (years old)		
				0-12	12-19	p-value^a^	Total			0-12	12-19	p-value^a^
				Mean (SD)	Mean (SD)		n (%)			n (%)	n (%)	
General	2.242 (100.0)	6.0 (3.5)		5.6 (3.3)	7.2 (4.0)	<0.001	53	21 (39.6)		8 (38.1)	13 (61.9)	0.004
Sex												
Female	1,387	6.1 (3.5)	0.011	5.6 (3.2)	7.5 (3.7)	<0.001	34 (64.1)	14 (41.2)	0.757	5 (35.7)	9 (64.3)	0.035
Male	855	7.8 (3.6)		5.6 (3.4)	6.5 (5.7)	0.007	19 (35.9)	07 (36.8)		3 (42.9)	4 (57.1)	0.129^b^
Type of PKU												
Classic	1,659	6.7 (3.7)	<0.001	6.4 (3.5)	7.5 (4.0)	<0.001	39 (73.6)	13 (33.3)	0.118	2 (15.4)	11 (84.6)	0.002^b^
Mild	583	4.0 (2.0)		4.0 (2.0)	3.8 (1.1)	0.708	14 (26.4)	08 (57.1)		6 (75.0)	2 (25.0)	0.473^b^
Family minimum wage (MW)												
Less than 1 MW	892	6.7 (4.0)	<0.001	6.0 (3.4)	8.2 (4.6)	<0.001	23 (48.9)	06 (26.1)	0.159	1 (16.7)	5 (83.3)	0.069^b^
1 MW or more	1,088	5.6 (3.1)		5.5 (3.2)	6.1 (2.5)	0.010	24 (51.1)	11 (45.8)		5 (45.4)	6 (54.6)	0.023
Maternal age (in 2019)												
Until 39	1,099	5.5 (3.2)	<0.001	5.6 (3.3)	3.7 (1.0)	<0.001	27 (50.9)	9 (33.3)	0.340	7 (77.8)	2 (22.2)	0.103^b^
40 or more	1,143	6.4 (3.7)		5.5 (3.3)	7.6 (4.0)	<0.001	26 (49.1)	12 (46.1)		1 (8.3)	11 (91.7)	0.036^b^
Maternal schooling												
≤5 years	518	5.8 (3.1)	0.006	5.8 (3.4)	5.7 (2.7)	0.927	12 (25.5)	06 (50.0)	0.176	-	6 (100)	0.061^b^
>5 years	1,470	6.2 (3.7)		5.7 (3.3)	9.1 (4.5)	<0.001	35 (74.5)	10 (28.6)		6 (60.0)	4 (40.0)	0.221^b^
Place of residence												
Rural area	991	6.3 (3.6)	0.053	5.8 (3.3)	7.9 (4.2)	<0.001	23 (48.9)	08 (34.8)	0.627	3 (37.5)	5 (62.5)	0.179^b^
Urban area	1,000	5.8 (3.4)		5.3 (3.1)	7.1 (3.8)	<0.001	24 (51.1)	10 (41.7)		3 (30.0)	7 (70.0)	0.017^b^

SRTN: Reference Service for Neonatal Screening (*Serviço de Referência em Triagem Neonatal*); Apae: Association of Parents and Friends of Intelectually Disabled Individuals (*Associação de Pais e Amigos dos Excepcionais de Salvador*); SD: standard deviation; PKU: phenylketonuria; MW: minimum wage; ^a^p-values related to the comparison between age groups; ^b^Fisher’s exact test.

Short height was observed in 1/53 (1.9%) participants (10-year-old boy). Thinness, according to the BMI/H indicator, was observed in 2/53 (3.8%), of participants, of two girls, two and 12 years old. Overweight was found in 9/53 (17%), with six girls (four teenagers) and three boys (one teen).

Regarding body composition, regardless of the anthropometric measure used, in the group of children and adolescents classified as adequate, individuals up to 12 years old predominated, but without statistical significance (Table 3). For the mean serum Phe levels, there was a consistent and significant trend of increasing these levels between categories: low reserve, adequate and excess/above average (Table 3). The percentage of metabolic control with 70% or more of adequate Phe levels was decreasing according to the AMA indicator (linear trend chi-square; p=0.042), being 70% among those with low reserve and 18.5% among those with excess (Table 3).

**Table 3:** Anthropometric and body composition indicators in 53 children and adolescents with phenylketonuria, stratified by age group, serum phenylalanine levels and metabolic control, demographic and socioeconomic variables. SRTN APAE Salvador, 2019.

	n (%)	Age range (years old)			Serum of phenylalanine (mg/dL)		Metabolic control ≥70%	
		0-12	12-19	p-value	Mean (SD)	p-value	n (%)	p-value
		n (%)	n (%)					
BMI/H (n=53)				0.832		<0.001*	21 (39.6)	0.881*
Thinness	02 (3.8)	1 (50.0)	1 (50.0)		3.7 (2.1)^a^		1 (50.0)	
Eutrophy	42 (79.2)	27 (64.3)	15 (35.7)		5.9 (3.4)^b^	<0.001^a.b^	17 (40.5)	
Excess	09 (17.0)	5 (55.6)	4 (44.4)		6.9 (3.9)^c^	<0.001^a.c/b.c^	3 (33.3)	
Body composition								
TST/H (n=52)				0.714		<0.001*	20 (38.5)	0.430*
Low reserve	13 (25.0)	9 (69.2)	4 (30.8)		4.9 (3.0)^a^		6 (46.1)	
Adequate	33 (63.5)	20 (60.6)	13 (39.4)		6 (3.5)^b^	<0.001ª^.b^	13 (39.4)	
Excess	06 (11.5)	3 (50.0)	3 (50.0)		8.4 (3.6)^c^	<0.001^a.c/b.c^	1 (16.7)	
SS/H (n=52)				0.465		<0.001*	21 (40.4)	
Low reserve	10 (19.2)	7 (70.0)	3 (30.0)		5 (3.2)^a^		4 (40.0)	0.884*
Adequate	33 (63.5)	19 (57.6)	14 (42.4)		6 (3.5)^b^	<0.001^a.b^	14 (42.4)	
Excess	09 (17.3)	7 (77.8)	2 (22.2)		6.4 (3.0)^c^	<0.001^a.c^	3 (33.3)	
TST+SS (n=49)				0.490		<0.001*	19 (38.8)	0.808**
Low reserve	13 (26.5)	9 (69.2)	4 (30.8)		5.1 (3.1)^a^		6 (46.1)	
Adequate	30 (61.2)	18 (60.0)	12 (40.0)		5.9 (3.2)^b^	<0.001^a.b^	11 (36.7)	
Excess	06 (12.3)	5 (83.3)	1 (16.7)		6.5 (3.2)^c^	<0.001^a.c/b.c^	2 (33.3)	
AMA (n=52)				0.134		<0.001*	20 (38.5)	0.042**
Low reserve	10 (19.2)	4 (40.0)	6 (60.0)		5.1 (2.7)^a^		7 (70.0)	
Adequate	31 (59.6)	19 (61.3)	12 (38.7)		6.3 (3.6)^b^	<0.001^a.b^	11 (35.5)	
Above average	11 (21.2)	9 (81.8)	2 (18.2)		6.1 (4.0)^c^	<0.001^a.c^	2 (18.2)	

SRTN: Reference Service for Neonatal Screening (*Serviço de Referência em Triagem Neonatal*); Apae: Association of Parents and Friends of Intelectually Disabled Individuals (*Associação de Pais e Amigos dos Excepcionais de Salvador*); SD: standard deviation; BMI: body mass index; TST: tricipital skinfold thickness; SS: subscapular skinfold; AMA: arm muscle area. *indicates p-value for ANOVA for comparison between the three categories of each anthropometric measure; ^a,b^indicates comparison between category A *versus* category B; ^a,c^indicates comparison between category A versus category C; ^b,c^indicates comparison between category A *versus* category C; **Chi-square of linear trend.

For adiposity rates, 1/52 (1.9%) individual was classified as having high WC, and 5/48 (10.4%), increased AbC. These participants had higher mean serum levels of Phe [standard deviation] (7.77 [4.17] *versus* 5.72 [3.18]mg/dL; p<0.001).

## DISCUSSION

To the best of our knowledge, this is the first Brazilian study to describe the metabolic control of children and adolescents with PKU in the Northeast of the country. We observed that almost 60% of the serum Phe dosages were within the recommended limits of adequacy,^19^ significantly higher among adolescents, reaching more than 68% of the dosages performed. Unlike other studies in our country, which consider serum Phe dosages in the past 12 months to assess metabolic control,^11^ ^,^ ^12^ in the present paper, the dosages from the last five years were considered. A total of 2,242 dosages (dosage average of 8.4 patient/year) was used for adequacy analyzes.

Additionally, we proposed a new way of defining adequacy, considering as the presence of at least 70% of dosages within the limit of adequacy recommended for age the “target” of good metabolic control. This criterion is adopted in the Guidelines of the Brazilian Diabetes Society to consider good glycemic control in diabetic individuals.^20^ Based on this, adolescents aged 12-19 had better metabolic control when compared to those aged 0-12 (65 *versus* 24.2%). In other countries, findings of worse metabolic control in adolescence are described, diverging from that from our study.^4^

In Brazil, in 94 adolescents between 10 and 20 years old with neonatal PKU in the Southeast of the country, 53.2% of Phe dosages were adequate, considered like this when the average of dosages were in the range between 2-10 mg/dL over the last 12 months.^11^ This finding is close to that of the present study in terms of the magnitude of metabolic control. However, two aspects must be highlighted. First, Camatta et al.^11^ consider the same cutoff point for Phe levels for adolescents aged 10-12 and older than 12, a fact that may have increased the frequency of adequacy, because the Brazilian protocol recommends a lower cut for patients aged 0-12 (2-6 mg/dL).^19^ Secondly, if we use the same criteria (mean of the last 12 months and limits of 2-10 ­mg/­dL for ages 10 years old and older), adequate metabolic control in 90% of individuals between 10 and 19 years old would be obtained (data not shown). This can be explained by the large number of Phe dosages between 6-8 mg/dL obtained within the range of 10-12 years old, which would be considered adequate for the recommended thresholds within 12-19 years old.

In the Southern region, of 27 patients with mild and classic PKU aged 6-25 and mostly with a late diagnosis (16/27; 59%), 48% of adequate metabolic control was described, using as the criteria the mean of at least three in up to 12 dosages of Phe over the last 12 months of up to 6 and 10 mg/dL, respectively, for individuals up to 12 years old and above that age.^10^ Also in this region, 84 phenylketonuric children and adolescents aged 2.4-19.9 were studied,^12^ using the average of the last three Phe dosages within the limits recommended by the Brazilian Ministry of Health as a suitability criterion.^19^ These authors described overall adequacy percentage of 65.5%, with no stratification by age group.^12^

The present study included individuals with early diagnosis and treatment, characteristics that probably decrease the dietary variations of the group, favor better metabolic control and low frequency of nutritional deficits observed. Late-treated patients may have different degrees of dependence to eat, depending on the magnitude of neurological sequelae, in addition to difficulty in modifying their pre-treatment eating habits. This probably has an effect on metabolic control^21^ and may explain part of the differences between studies conducted in Brazil.

Previous studies have reported that patients with PKU with no diet control are at increased risk of becoming overweight.^5^ ^,^ ^6^ In our study, this finding was found in 17% of patients, with 20% among adolescents. Similarity was observed in relation to metabolic control, in comparison with the groups with eutrophy and thinness. Excess body fat was less frequent in the sample studied, according to the TST and SS indicators alone or added together, with no statistical differences between groups when assessing metabolic control. In addition, the present investigation found a low frequency of central adiposity, a protective factor for metabolic syndrome in these patients.

Research carried out in the South and Southeast of Brazil described higher percentages of overweight, which were 28.5,^12^, 22^10^, and 19.1%^11^, respectively. Recent data on the nutritional transition in Brazil show that there are important regional differences in the magnitude of overweight^22^ and that factors such as family income and place of residence^23^ influence these indicators. Although controversial, overweight in the population with PKU seems to tend to follow the course of the general population.^24^ Similarly, other national studies also found no differences in the frequency of adequacy of Phe dosages between the groups of overweight and normal weight BMI.^10^ ^,^ ^11^ ^,^ ^12^

Other studies, however, found a relation between Phe levels and body fat. A study with 30 North American children and adolescents identified 40% of overweight and a positive relation between Phe levels and body fat, based on the last 12 dosages. Comparing to a control group without the disease, the author suggests that there is a difference in body composition, influenced not only by diet, but also by other factors, such as genetic and those related to physical activity.^5^

We found most of those surveyed with adequate or above average muscle tissue reserve, a finding that may reveal satisfactory nutritional prescription and adequate consumption of the Phe-free metabolic formula, which has efficient bioavailability and is the main source of protein (75-85%) of their diet. However, AMA classified as above average or adequate was associated with worse percentages of metabolic control. This fact may also suggest, in addition to good adherence to the use of metabolic formula, possible transgressions, with consumption of prohibited proteins. Evaluation of 37 people with PKU between 5 and 18 years old revealed a significant negative correlation between body fat percentage and protein and amino acid formula intake.^25^

Although not significant, individuals from families with income of one or more minimum wages (MW) had better metabolic control, which may be related to greater access to industrialized hypoproteinic foods and, consequently, greater variation in their diet. Unlike European countries and North America, there are no government subsidies for the purchase of these foods in Brazil. A study conducted in Spain concluded that the high cost of the diet and the difficulty of acquiring special hypoprotein foods have an effect on poor adherence to treatment.^26^

Similarly, there was no statistically significant association between maternal education and metabolic control. However, among mothers/caregivers who studied up to the fifth year of elementary school, 50% of patients were adequate, whereas only 28.6% of those whose caregivers had more education were also adequate, diverging from a previous study.^22^ The data is unusual, considering that education has a strong relationship with income. We assume that a portion of these individuals is also included in the group with an income above an MW, as some families receive the benefit of continued provision (*benefício de prestação continuada* - BPC) and other social benefits, such as *Bolsa Família*, so that the variable income may be interfering in this result. Another hypothesis is that the less educated mother not frequently enters the labor market, offering greater dedication to the child, which includes greater surveillance of consumption and availability to prepare low-cost hypoprotein foods with guidance from the health team. This is because a singularity of the studied population is the culinary workshop as part of interdisciplinary monitoring, a place specially designed for the preparation of hypoproteinic foods, with less cost.

The present investigation has the limitation of not having evaluated dietary data. The majority of the examined population does not reside in the municipality of treatment and travels long distances to receive it, which makes the application of the 24-hour food record impossible. In addition, many patients and family members have difficulty keeping a three-day food record. Although there is no consensus, the nature of dietary treatment and the bioavailability of L-amino acids in the metabolic formula are likely to have effects on growth and body composition in this population.^5^ ^,^ ^6^ ^,^ ^24^ ^,^ ^27^ In addition, information about the food routine depends on the report of the individual and/or their caregivers, who often omit food transgressions to the health team.

Despite the limitations, unlike the Southern and Southeastern regions of Brazil and European countries and the United States, the study population is predominantly low-income and, certainly, has limited access to industrialized hypoprotein foods. Therefore, blood levels of Phe are the most accurate indicators available to investigate adherence to treatment. Serum Phe levels reflect recent food consumption, and there are no tests that can estimate their fluctuations. Thus, returning to the diet a few days before the test is sufficient to normalize Phe levels, masking inadequate metabolic control.

In conclusion, adequate metabolic control was observed in almost 60% of the patients studied, with a significantly higher percentage among adolescents aged 12-19. Overweight was less frequent than that observed in other national studies and was not associated with worse metabolic control. The low prevalence of anthropometric changes suggests that regular nutritional and clinical follow-up contributes to better monitoring and early interventions for adaptation to anthropometric evolution.
